# Autophagy activated via GRP78 to alleviate endoplasmic reticulum stress for cell survival in blue light-mediated damage of A2E-laden RPEs

**DOI:** 10.1186/s12886-019-1261-4

**Published:** 2019-12-10

**Authors:** Jingyang Feng, Yuhong Chen, Bing Lu, Xiangjun Sun, Hong Zhu, Xiaodong Sun

**Affiliations:** 1 Department of Ophthalmology, Shanghai General Hospital (Shanghai First People’s Hospital), Shanghai Jiao Tong University School of Medicine, No.100 Hai Ning Road, Shanghai, 200080 China; 2 Shanghai Engineering Center for Visual Science and Photomedicine, No.100 Hai Ning Road, Shanghai, 200080 China; 3 Shanghai Jiao Tong University School of Biology and Agriculture, Shanghai, China; 4 Shanghai Key Laboratory of Fundus Disease, No.100 Hai Ning Road, Shanghai, 200080 China

**Keywords:** Autophagy, Endoplasmic reticulum stress, Glucose-related protein 78, Retinal pigment epithelium, N-retinylidene-N-retinylethanolamine

## Abstract

**Background:**

Retinal pigment epithelium cells (RPEs) are critical for maintaining retinal homeostasis. Accumulation of age-related lipofuscin, N-retinylidene-N-retinylethanolamine (A2E), makes RPEs vulnerable to blue light-mediated damage, which represents an initial cause of some retinal degenerative diseases. This study investigated the activation of autophagy and the signaling pathway involved in glucose-related protein 78 (GRP78) induced autophagy in blue light-mediated damage of A2E-laden RPEs. In addition, we explored whether autophagy could play a protective role by alleviating endoplasmic reticulum (ER) stress to promote RPEs survival.

**Methods:**

RPEs were incubated with 25 μM A2E for 2 h and exposed to blue light for 20 min. The expression of ER stress-related apoptotic proteins, CHOP and caspase-12, as well as autophagy marker LC3 were measured by western blot analysis. Autophagosomes were observed by both transmission electron microscopy and immunofluorescence assays. GRP78 interference performed by short hairpin RNA (shRNA) was used to identify the signaling pathway involved in GRP78 induced autophagy. Cell death was assessed using TUNEL analysis.

**Results:**

Treatment with A2E and blue light markedly increased the expression of ER stress-related apoptotic molecules CHOP and caspase-12. The activation of autophagy was recognized by observing autophagosomes at ultrastructural level. Additionally, punctate distributions of LC3 immunofluorescence and enhanced conversions of LC3-I to LC3-II were found in A2E and blue light-treated RPEs. Moreover, GRP78 interference reduced AMPK phosphorylation and promoted mTOR activity, thereby downregulating autophagy. In addition, the inhibition of autophagy made RPEs vulnerable to A2E and blue light damage. In contrast, the autophagy inducer rapamycin alleviated ER stress to promote RPEs survival.

**Conclusions:**

GRP78 activates autophagy via AMPK/mTOR in blue light-mediated damage of A2E-laden RPEs in vitro. Autophagy may be a vital endogenous cytoprotective process to alleviate stress for RPEs survival in retinal degenerative diseases.

## Background

The Retinal pigment epithelium (RPE) is a single layer of cells located between the retinal photoreceptors and choriocapillaris layer. RPE cells (RPEs) play multiple essential roles in sustaining function and survival of the overlying photoreceptors by comprising the outer blood-retinal barrier, maintaining the retinoid cycle, providing nutritional factors, and phagocytosing photoreceptor outer segment (POS) [1]. Along with the aging, a large amount of lipofuscin derived from ingestion of POS accumulates in RPEs, which is an initial cause of RPE damage in some retinal degenerative disorders such as age-related macular degeneration (AMD) [2, 3].

N-retinylidene-N-retinylethanolamine (A2E) is the main hydrophobic fluorophore of RPE lipofuscin which is generated from all-trans-retinal [4]. A2E plays the role of a photosensitizer that generates singlet oxygen and peroxide upon exposure to blue light [5]. Our previous study confirmed that A2E and blue light stimuli caused cytotoxicity in RPEs. Moreover, these RPEs exhibited the increase of two major endoplasmic reticulum (ER) stress molecules, glucose-related protein 78 (GRP78) and C/EBP homologous protein (CHOP), suggesting the activation of ER stress in blue light-induced damage of A2E-laden RPEs [6].

Autophagy is a highly conserved “self-eating” mechanism in eukaryotic cells for degrading and recycling cytoplasmic components via the lysosomal degradation pathway [7]. The initiation of autophagic process includes the formation of phagophores which generally expand into double membrane vacuoles termed autophagosomes. Autophagosomes sequester cellular materials as cargo and then fuse with lysosomes to degrade the contents [8]. Many forms of biochemical and pathological stress can induce autophagy. The proper activation of autophagy can remove harmful cellular components and damaged organelles to restore intracellular homeostasis [9]. However, the age-related impairment of autophagy can cause cells to become overwhelmed by the aggregation of damaged proteins and organelles, which has been reported to be associated with many degenerative and age-related disorders such as AMD [10, 11].

GRP78 as a protective molecular chaperone initiates the unfolded protein response (UPR) to help refold proteins during ER stress [12]. In recent years, it has been recognized to be involved in stress-induced autophagy regulation [13]. Thus, we speculate that GRP78 may regulate the autophagic pathway under ER stress in blue light-induced damage of A2E-containing RPEs. In current study, we found that the activation of ER stress-related cell death caused by A2E and blue light damage in RPEs. GRP78-autophagy pathway is a potential mechanism for RPEs survival under ER stress. Our results high light the importance of GRP78 in regulating autophagy and suggest that it could be a possible strategy for treating RPE-derived retinal degenerative disorders.

## Methods

### RPEs culture

ARPE-19 cells (American Type Culture Collection, Manassas, VA, USA) at passages 12, absent of endogenous A2E were cultivated under 37 °C humidified 5% CO_2_ circumstance in Dulbecco’s modified Eagle’s/ Ham’s F_12_ medium (DMEM/F12; Invitrogen, Grand Island, NY, USA) containing 10% fetal bovine serum (FBS), 100 U/ml penicillin and 100 μg/ml streptomycin, as previously described [6]. The RPEs were delivered in different culture plates based on each experiment’s requirement. When achieving confluence, RPEs were transferred to serum-free medium for another 24 h before accepting treatments.

### A2E synthesis and treatment paradigm

A2E was prepared from 100 mg all-trans-retinal (Sigma Aldrich, St. Louis, MO, USA) and 9.5 mg ethanolamine in 2 ml ethanol as previously described [14]. A2E was stored in dimethyl sulfoxide (DMSO) at 25 mM in the dark under − 80 °C.

Confluent RPEs were cultivated with 25 μM A2E in medium for 2 h. Then, extracellular A2E was washed off. After A2E intaking, RPEs were illuminated by 460 ± 20 nm wavelength light (4000 lx; OSRAM GmbH, Augsburg, Germany) for 20 min, as previously described [6]. To regulate autophagy, RPEs were pretreated with 5 mM 3-methyladenine (3-MA) or 10 nM rapamycin for 2 h before A2E loading and illumination.

### Western blot analysis

RPEs grown in 90-mm diameter dishes were harvested in lysis buffer after indicated treatments. After centrifugation at 4 °C, supernatants containing proteins were collected and reserved at − 80 °C until use. A Bio-Rad protein assay (Bio-Rad, Hercules, CA, USA) was used for protein immunoblot analysis. The protein was separated by sodium dodecyl sulfate polyacrylamide gel electrophoresis (SDS-PAGE). After transfer of proteins, membranes were blocked and incubated overnight at 4 °C with the primary antibodies including GRP78 monoclonal antibody (1:1000; Epitomics Inc., Burlingame, CA, USA), CHOP polyclonal antibody (1:200; Santa Cruz Biotechnology, Inc., Santa Cruz, CA, USA), caspase-12 polyclonal antibody (1:200; Santa Cruz Biotechnology Inc., Santa Cruz, CA, USA), LC3 monoclonal antibody (1:1000; Cell Signaling Technology, Danvers, MA, USA), AMPK monoclonal antibody (1:1000; Cell Signaling Technology, Danvers, MA, USA), phospho-AMPK monoclonal antibody (1:1000; Cell Signaling Technology, Danvers, MA, USA), mTOR monoclonal antibody (1:1000; Cell Signaling Technology, Danvers, MA, USA), or phospho-mTOR monoclonal antibody (1:1000; Cell Signaling Technology, Danvers, MA, USA). Secondary antibody conjugated with horseradish peroxidase (IgG-HRP; 1:2000; Cell Signaling Technology, Danvers, MA, USA) and enhanced chemiluminescence kit (ECL; GE Healthcare Life Sciences, Buckinghamshire, UK) were used to detect binding.

### Transmission electron microscopy analysis

The treated RPEs were washed and fixed in 0.1 M cacodylate buffer (pH = 7.4) containing 2.5% glutaraldehyde and 2% paraformaldehyde for 1 h at room temperature. The samples were fixed for 1 h in 1% osmium tetroxide, prior to dehydration in a graded ethanol series and embedded in epon 812 resin. Then, ultra-thin sections containing cells were block-stained with uranyl acetate and lead citrate; and examined with a transmission electron microscope (CM120; Philips, Eindhoven, Netherlands). We evaluated a minimum of 6 images per sample.

### Immunofluorescent analysis

After completion of respective treatments, RPEs were washed with phosphate-buffered saline (PBS) and fixed in 4% paraformaldehyde for 20 min. The cells were then permeabilized with 0.1% Triton X-100 for 15 min and blocked for 1 h at room temperature. The cells were subsequently incubated with primary LC3 antibody in blocking solution at 4 °C overnight. The next day, cells were washed and then incubated with an Alexa Fluor 555-conjugated secondary antibody (IgG; 1:1000, Cell Signaling Technology, Danvers, MA, USA) for 1 h at room temperature. The cells were briefly stained with 4′,6′-diamidino-2-phenylindole (DAPI) to visualize the nuclei. The fluorescence of LC3 punctate was detected by a confocal laser microscope (FV1000; Olympus, Japan) with a 540 nm band-pass filter after excitation at 488 nm. DAPI was excited at 568 nm and visualized with 670 nm band-pass filters. Autophagosomes were quantified by counting the number of LC3 fluorescent puncta in per RPE cell.

### Short hairpin RNA interference of GRP78

Lentiviral vectors carrying a short hairpin RNA (shRNA) targeting GRP78 or negative control shRNA were produced by GenePharma Co. Ltd. (Shanghai, China). The sequence of shRNA targeting GRP78 was 5′-GGAACTGGAAGAAATTGTTCA-3′. The sequence of shRNA used as a negative control was 5′-TTCTCCGAACGTGTCACGT-3′. Stably transfected RPEs were selected and obtained as previously described [6].

### TUNEL analysis

After the indicated treatments, RPEs were washed thrice with PBS and fixed for 10 min with 4% paraformaldehyde at room temperature. After fixation, TUNEL staining was performed for 30 min at 37 °C in the dark. Then, cells were washed with PBS three times and examined under a confocal laser microscope. The number of death cells, which were identified as having TUNEL positive nuclei, in RPEs was counted.

### Statistical analysis

These assays were performed at least three times. All quantitative data was recorded as mean ± SD. SPSS 21.0 version (IBM SPSS, Chicago, IL, USA) was used to evaluate difference by Student’s t-test or one-way analysis of variance (ANOVA). A *p*-value below 0.05 was considered statistically significant.

## Results

### Induction of ER stress-related cell death in blue light-mediated damage of A2E-laden RPEs

**Fig. 1:**
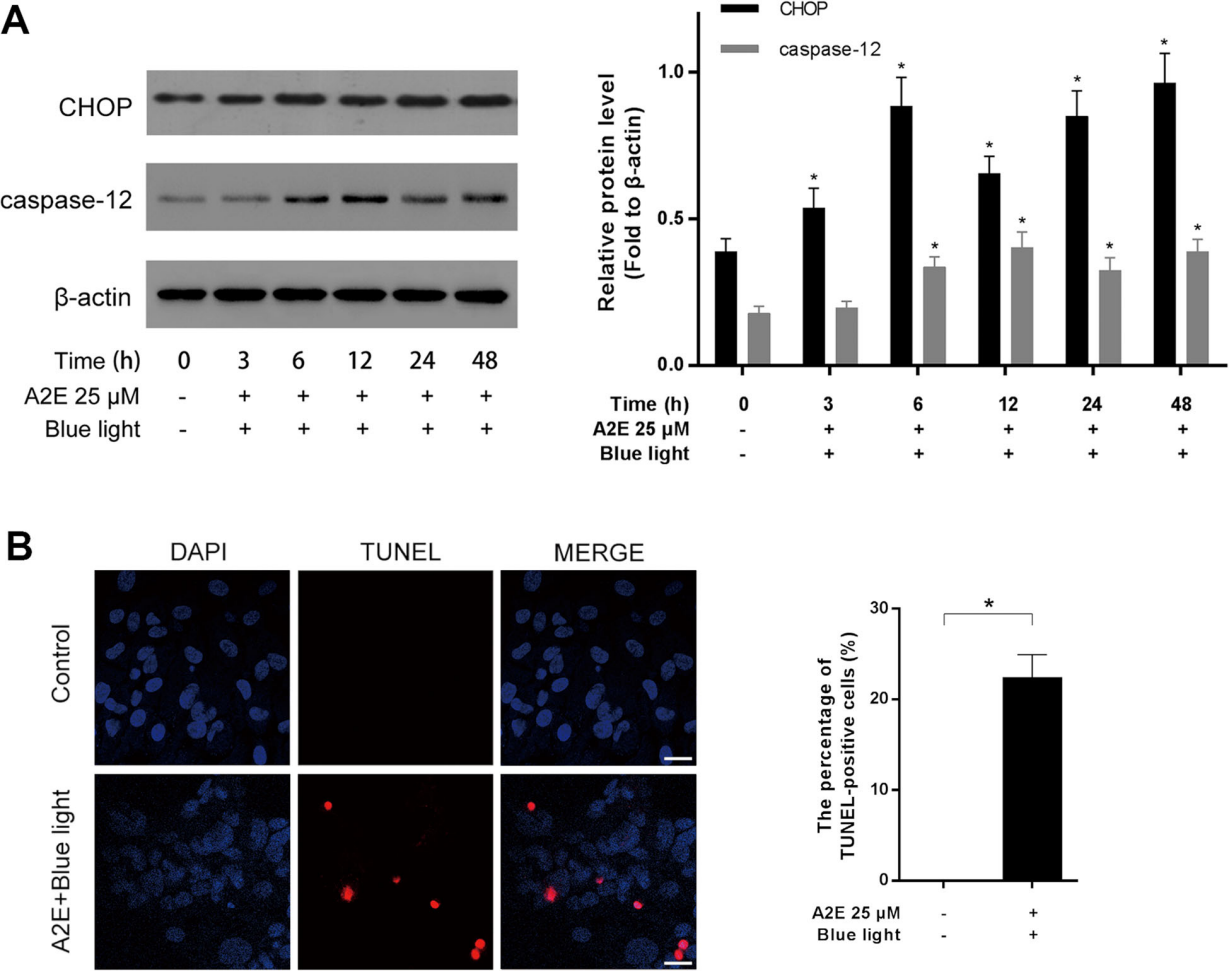
Induction of ER stress-related cell death in blue light-mediated damage of A2E-laden RPEs. **a**: RPEs were incubated with 25 μM A2E for 2 h, then exposed to blue light for 20 min. At indicated time points, CHOP and caspase-12 protein levels were detected by western blot analysis. β-actin was used as the internal control. Bars represent the mean ± SD from three independent experiments (*n* = 3, *p < 0.05 versus control). **b**: TUNEL analysis was used to measure RPEs death at 12 h after A2E and blue light treatment (scale bar, 10 μm). The cell death rates are reported as percentage of TUNEL positive cells (*n* = 3, *p < 0.05).

**Fig. 2:**
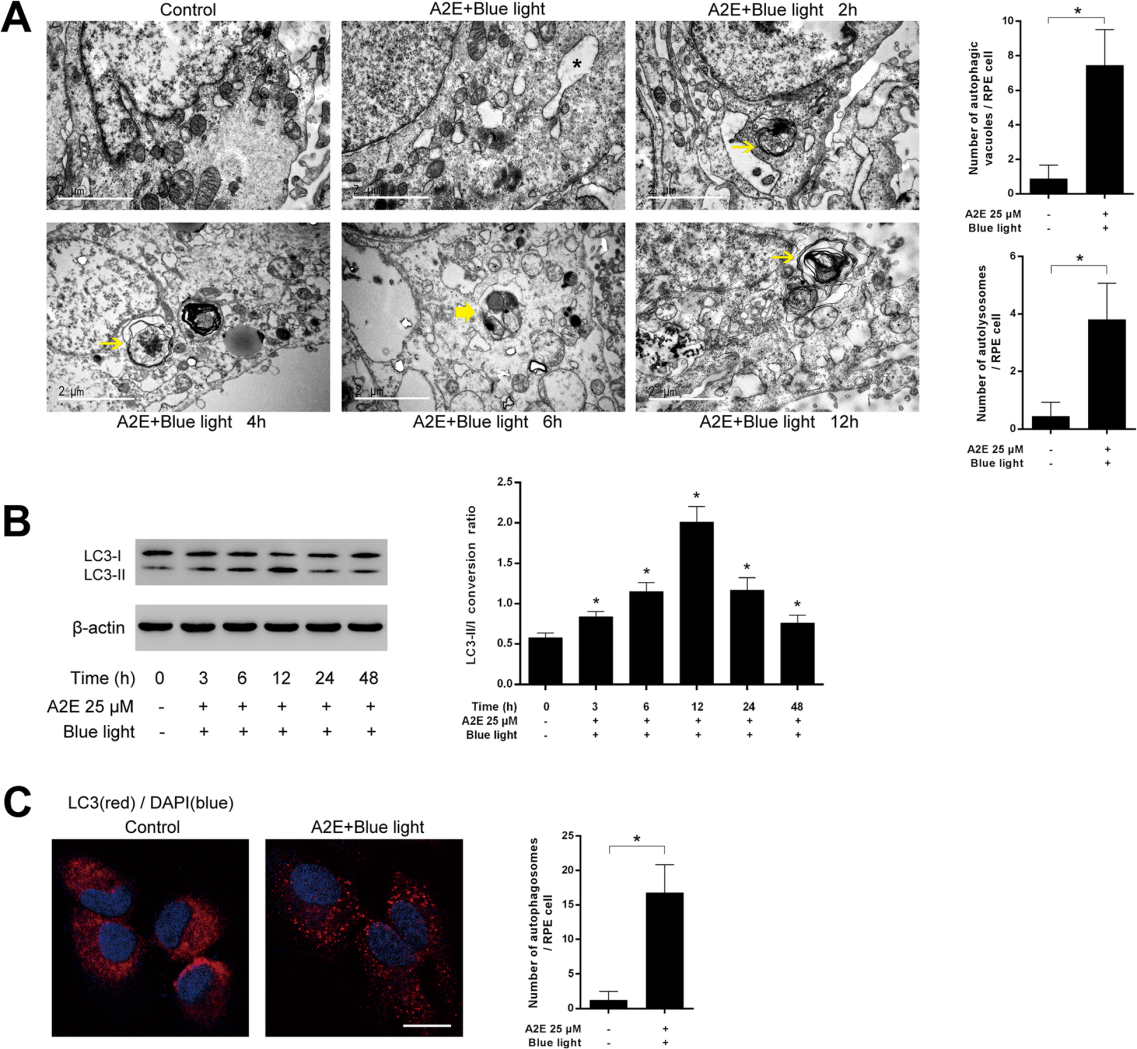
Autophagy activation in blue light-mediated damage of A2E-laden RPEs. **a**: Representative TEM images of RPEs at indicated time points after treated with 25 μM A2E and blue light exposure (Scale bar, 2 μm). The formation of autophagic vacuoles (yellow arrows) and autolysosomes (bold arrow) was observed. Some ER exhibited a disorganized structure with a swollen appearance (asterisk). The number of autophagic vacuoles and autolysosomes at 12 h after treatment was counted in per RPE cell from three independent experiments (*n* = 3, *p < 0.05). **b**: Western blot analysis of LC3 was performed at indicated time points after treatment. The results are reported as the conversion of LC3-I to LC3-II. Bars represent the mean ± SD from three independent experiments (*n* = 3, *p < 0.05 versus control). **c**: Autophagosome formation in RPEs was visualized by LC3 immunofluorescence. LC3 appeared a diffuse and cytosolic pattern in control RPEs. An increased LC3 puncta was observed at 12 h after A2E and blue light treatment. (scale bar, 10 μm). The number of autophagosomes was obtained by counting LC3 fluorescent puncta in RPEs from three independent experiments (*n* = 3, *p < 0.05).

RPEs were incubated with A2E and exposed to blue light. To confirm that ER stress-related cell death took place in A2E and blue light treated RPEs, we analyzed two relevant molecules, CHOP and caspase-12, by western blot. CHOP protein level showed a quick ascent at 6 h and reached another peak at 48 h after treatment. For caspase-12 expression, the protein level gradually increased along with the experimental period and reached a peak at 12 h after treatment (Fig. 1a). TUNEL analysis was used to detect RPEs death. Twelve hours after A2E and blue light treatment, RPEs suffered significant cell death compared with the control group (Fig. 1b). Additionally, via a transmission electron microscopy, we observed that the ER exhibited a swollen appearance in A2E-laden RPEs after blue light illumination (Fig. 2a), which indicated the damage of ER. These results reveal that ER stress-related cell death might be activated in blue light-induced damage of A2E-containing RPEs.

### Autophagy activation in blue light-mediated damage of A2E-laden RPEs

To study whether autophagy is activated in A2E-containing RPEs after blue light exposure, we initially monitored autophagic vacuole formation via a transmission electron microscopy (TEM). From 2 to 12 h after treatment with A2E and blue light, we observed the formation of autophagic vacuoles which were defined as double-membrane vacuoles containing cytoplasmic materials at ultrastructural level (Fig. 2a). Autolysosomes which usually have only one limiting membrane and contain electron-dense amorphous contents increased in A2E and blue light treated RPEs. In contrast, we seldomly found initial or late autophagic vacuoles in control RPEs. Next, western blotting analysis was performed to examine the expression of autophagic marker LC3 at indicated time points. The conversion of LC3-I to LC3-II in RPEs exposed to A2E and blue light significantly increased from 3 to 48 h and reached a peak at 12 h after treatment compared with that in control cells (Fig. 2b). Furthermore, immunofluorescence analysis with LC3 antibody was performed. In control cells, the distribution of LC3 exhibited a diffuse and cytosolic pattern. Twelve hours after A2E and blue light exposure, LC3 exhibited a punctate distribution in RPEs, and the number of characteristic fluorescent puncta significantly increased, which was evidence of enhanced the formation of autophagosomes (Fig. 2c). Taken together, these results suggest increased autophagic activity in blue light-induced damage of A2E-containing RPEs.

### GRP78 regulates autophagy via AMPK/mTOR pathway in A2E and blue light-treated RPEs

**Fig. 3:**
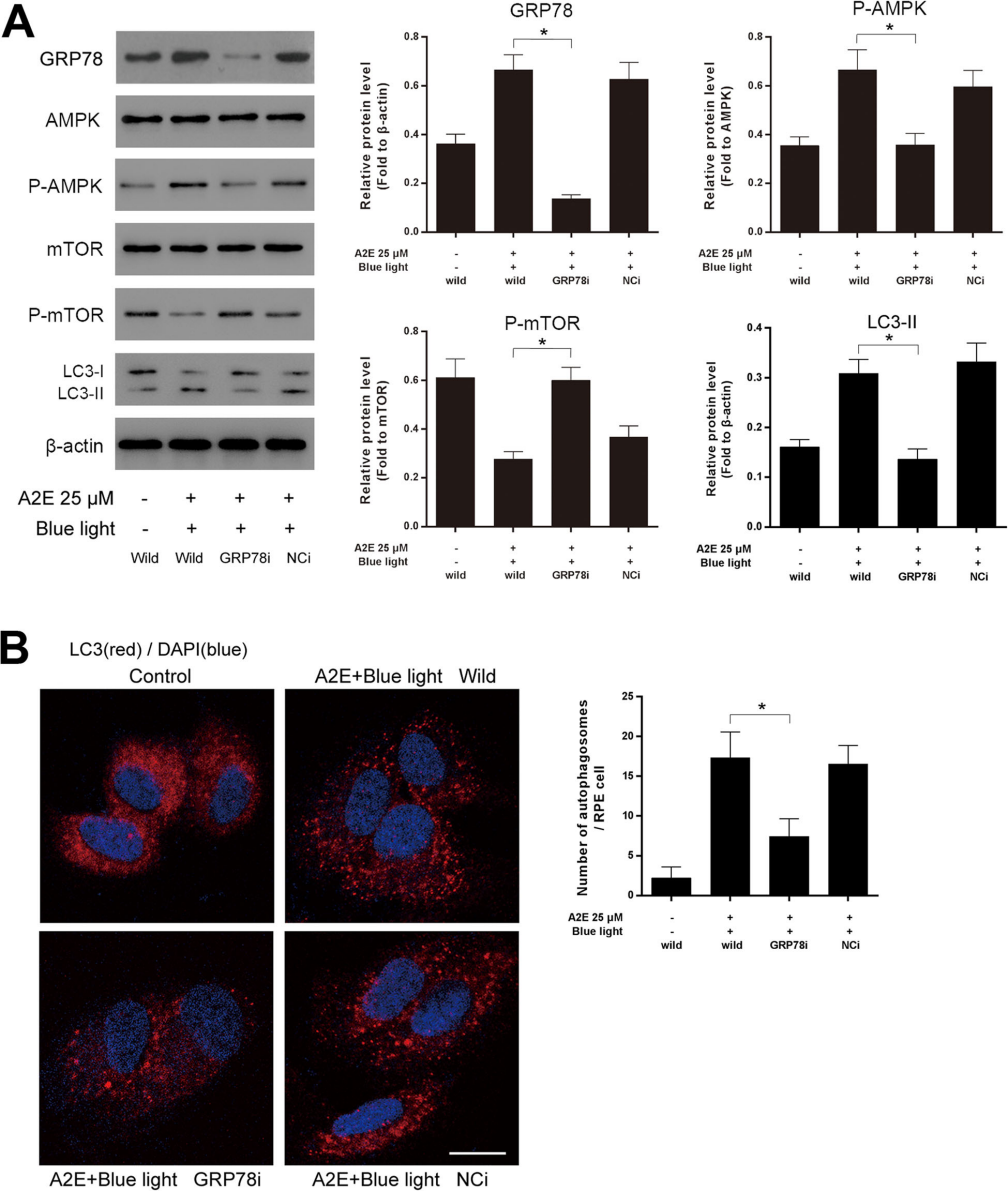
GRP78 regulates autophagy via AMPK/mTOR in A2E and blue light-treated RPEs. **a**: Wild RPEs and RPEs received GRP78 shRNA (GRP78i) or negative control shRNA (NCi) were treated with 25 μM A2E and blue light exposure. Cells were collected 12 h later. GRP78, AMPK, mTOR, p-AMPK, p-mTOR and LC3 protein levels were assessed by western blot analysis. Bars represent the mean ± SD from three independent experiments (*n* = 3, *p < 0.05). **b**: LC3 immunofluorescence was detected at 12 h after treatment. In wild RPEs or in negative vector group, LC3 revealed an increased dots distribution. GRP78 shRNA attenuated the formation of LC3 puncta in A2E and blue light-treated RPEs (scale bar, 10 μm). The number of autophagosomes was obtained by counting LC3 fluorescent puncta in RPEs from three independent experiments (*n* = 3, *p < 0.05).

To confirm whether GRP78 participates in the signaling required for autophagy regulation after A2E and blue light damage, we pretreated RPEs with GRP78 shRNA. In the GRP78-deficient group, the GRP78 expression was significantly inhibited, and the level of LC3-II was evidently decreased compared with that in wild cells at 12 h after A2E and blue light exposure. Meanwhile, GRP78 interference significantly reduced the phosphorylation of AMPK and promoted the phosphorylation of autophagy negative regulator mTOR (Fig. 3a). Furthermore, LC3 immunofluorescence analysis was applied to detect autophagy formation. In wild RPEs or in negative vector group, we observed an increase in the punctate distribution of LC3 in RPEs cytoplasm at 12 h after treatment with A2E and blue light. In contrast, we observed fewer LC3 puncta in the GRP78 shRNA group (Fig. 3b). Overall, we conclude that GRP78 plays a positive role in regulating autophagy via AMPK/mTOR signaling during ER stress in A2E-laden RPEs after blue light exposure.

### Autophagy alleviates ER stress for cell survival in blue light-mediated damage of A2E-laden RPEs

**Fig. 4:**
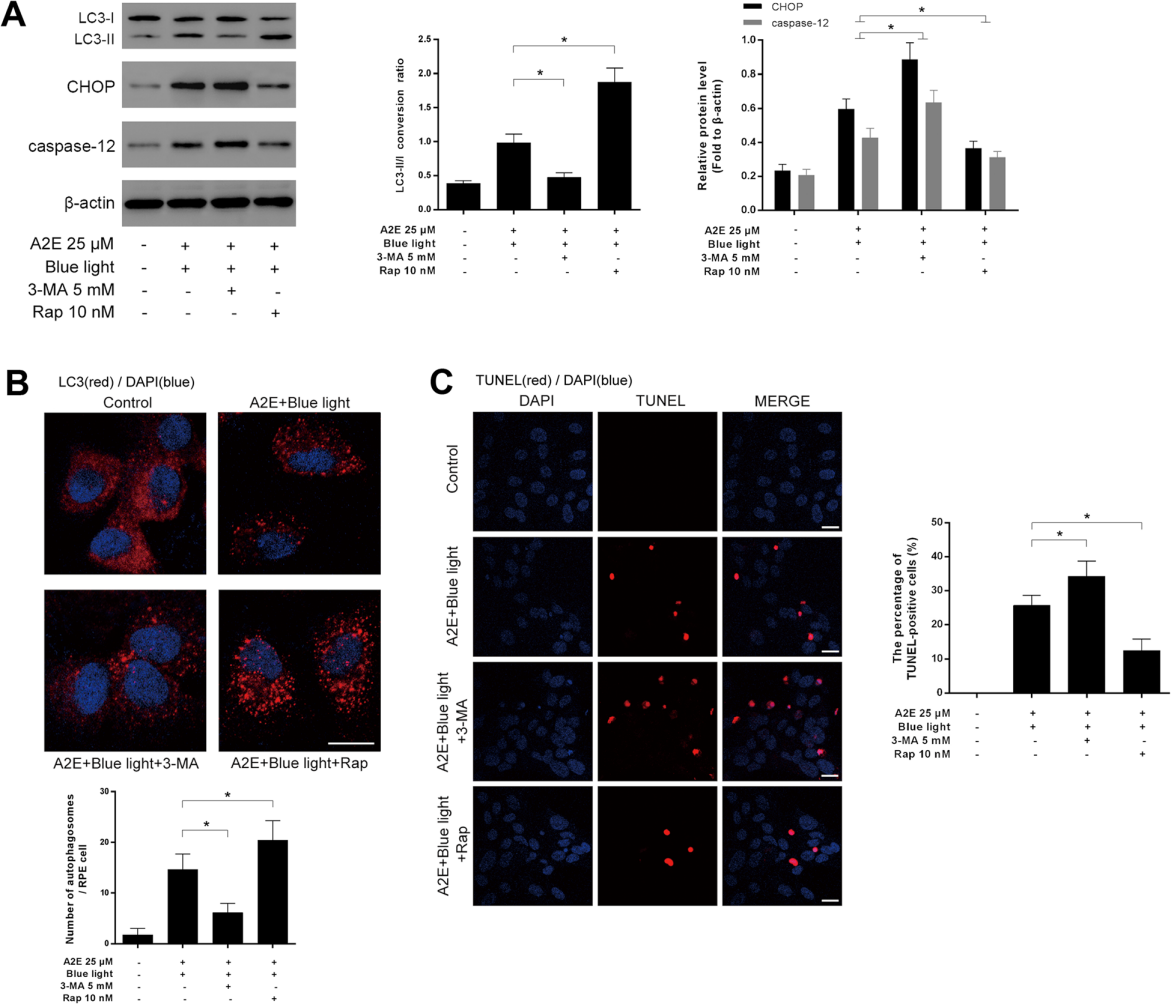
Autophagy alleviates ER stress for cell survival in blue light-mediated damage of A2E-laden RPEs. **a**: RPEs were preincubated with 10 nM rapamycin or 5 mM 3-MA for 2 h, then cultured with 25 μM A2E for 2 h and exposed to blue light for 20 min. Cells were collected 12 h later. Western blot was used to measure LC3, CHOP and caspase-12 protein levels. Bars represent the mean ± SD from three independent experiments (*n* = 3, *p < 0.05). **b**: The immunofluorescence of LC3 was measured by confocal laser microscopy. In rapamycin pretreated group, LC3 showed an increased punctate distribution. On the contrary, 3-MA attenuated LC3 dots formation (scale bar, 10 μm). The number of autophagosomes was obtained by counting LC3 fluorescent puncta in RPEs from three independent experiments (*n* = 3, *p < 0.05). **c**: Cell death in RPEs was measured by TUNEL analysis at 12 h after A2E and blue light treatment (scale bar, 10 μm). The cell death rates are reported as percentage of TUNEL positive cells (*n* = 3, *p < 0.05)

It is paradoxical that autophagy can contribute to protect cells but may also lead to cell damage. To clarify whether autophagy plays a protective or detrimental role, we pretreated RPEs with autophagy inducer rapamycin or inhibitor 3-MA respectively. Rapamycin pretreated group showed an increased conversion of LC3-I to LC3-II at 12 h after A2E and blue light exposure, while the ratio in 3-MA pretreated group was decreased compared with that in the non-pretreated group (Fig. 4a). The distribution and number of LC3 fluorescent puncta revealed a similar result (Fig. 4b). We also analyzed two ER stress-related apoptosis molecules, CHOP and caspase-12. Compared to non-pretreated group, 3-MA pretreated RPEs showed a dramatic increase in the expression of CHOP and caspase-12 at 12 h after A2E and blue light treatment. However, in rapamycin pretreated group, the CHOP and caspase-12 expression were at a lower level than none-pretreated group (Fig. 4a). Furthermore, TUNEL analysis was used to monitor cell death. In 3-MA pretreated group, RPEs suffered significant cell death at 12 h after A2E and blue light exposure compared with that in the non-pretreated group. In contrast, RPEs pretreated with autophagy inducer rapamycin were more resistant to A2E and blue light-induced cell death (Fig. 4c). Therefore, we deduced that autophagy plays a pivotal role in protecting against the ER stress-related cell death in blue light-mediated damage of A2E-containing RPEs.

## Discussion

In this study, we revealed the upregulation of ER stress-related apoptotic proteins and the activation of autophagy in blue light-mediated damage of A2E-containing RPEs. Mechanistically, we demonstrated that GRP78 could regulate autophagy via AMPK/mTOR signaling pathway during RPEs damage. Furthermore, induction of autophagy could act as a protective mechanism to alleviate ER stress for RPEs survival.

Along with human being’s age growing, bisretinoid compounds, such as lipofuscin, are deposited in RPEs, which has been recognized as a prominent feature of RPEs aging and is correlated with a variety of macular degenerative disorders such as Stargardt’s disease and AMD [15]. An analysis of extracts from human RPEs revealed that the major RPE lipofuscin is A2E [16]. In our previous study, compared to either 25 μM A2E or blue light treatment alone, combined treatment resulted in significant decrease in RPEs viability [6]. It has also been suggested that A2E can exhibit cytotoxicity by photochemical damage and is an initiator of blue light-mediated damage of RPEs [17]. Thus, we established an in vitro model of RPEs damage treated by both A2E and blue light for present study.

It was revealed that ER stress takes part in the blue light-mediated damage of A2E-laden RPEs [6]. This study further confirmed that two ER stress-related apoptotic proteins, CHOP and caspase-12, increased after treatment. Upon ER stress, GRP78 first dissociates from ER membrane to help destroyed proteins refold and degrade. However, excessive damaged proteins that exceed the capacity of restoration can lead to ER stress-related cell death [18]. Several current reports have also proposed that ER stress may be an important mechanism contributing to the damage of RPEs and pathogenesis of some macular degenerative diseases, especially AMD [19, 20]. Thus, understanding the process by which ER stress in RPEs is responded and alleviated may provide insight into potential therapeutic targets for diseases.

Autophagy serves as a cellular endogenous response to stress. Indeed, our present experiments observed increased formation of autophagosomes during ER stress in A2E and blue light-treated RPEs. The conversion of autophagy marker LC3-I to LC3-II elevated after treatment and peaked in the middle period. Accumulating data now have indicated that, besides nutrient depletion and hypoxia, ER stress have emerged as a novel autophagy inducer, which raises increasing attention [7]. However, little is known about how autophagy is regulated under ER stress in blue light-induced damaged of A2E-containing RPEs. In our study, while silent expression of GRP78, both autophagosomes formation and conversion of LC3-I to LC3-II decreased in A2E and blue light-treated RPEs. Moreover, downregulation of GRP78 could also lead to decrease phosphorylation of AMPK and promote the activity of p-mTOR. As a molecular goal keeper of autophagy, p-mTOR activation contributes to autophagy inhibition. In recent years, heat shock protein family including GRP78 has been delineated to be a novel regulator of autophagic pathway [21]. Considering its multifunctional roles in protein folding, degradation and cellular protection, GRP78 may be a key point of interaction between ER stress and autophagy. In addition, there is study showing that autophagy could be induced via PERK/eIF2a phosphorylation during ER stress in mammalian cells [22]. Also, the sirtuin 6 (SIRT6) pathway has been reported to be involved in the modulation of autophagy in RPEs [23]. Until now, it still remains exploration of which signaling pathway contributes the most in response to stress. At least, our results infer that GRP78/AMPK/mTOR pathway is one of important signaling pathways for autophagy regulation during ER stress in RPEs.

As a highly conserved process from yeast to mammals, autophagy plays a controversial role in regulating cell death and survival [24]. In this study, we revealed that rapamycin, as an upregulator of autophagy, led to alleviate A2E and blue light-induced damage to RPEs. In contrast, suppression of autophagy by 3-MA aggravated RPEs death. The protective role of autophagy has also been demonstrated experimentally in many different systems [25]. In a rat model of neonatal central nervous system hypoxia, autophagy was activated within the ischemic areas and reduced the cellular loss induced by the lack of oxygen [26]. Similarly, in animal models of chronic neurodegenerative diseases, activation of autophagy is correlated with increased cell survival [27]. However, how autophagy inhibits cell death remains to be elucidated. It is considered that autophagy can directly sequester pro-apoptotic factors such as caspases and promote their degradation [28]. In our experiments, we observed that increase of autophagy could decrease ER stress-related apoptotic markers, CHOP and caspase-12, in RPEs during A2E and blue light damage. Previous research also suggested that autophagy was critical for neuroprotection to sustain ER homeostasis [29]. Pancreatic β cells subjected to severe ER stress revealed an autophagic response, which countered ER expansion [30]. Upon ER stress, autophagy can act as a cytoprotective response to degrade excessive unfolded or misfolded proteins and damaged organelles.

## Conclusions

Our findings have revealed that GRP78 mediates activation of autophagy via AMPK/mTOR to alleviate ER stress-related cell death in blue light-induced damage of A2E-laden RPEs in vitro. However, the use of ARPE-19 cells in this study may exist some limitations, as in current understanding, ARPE-19 cells only partially recapitulate the features of human RPEs. Further exploration using primary human RPEs or in vivo models are needed. Since the damage of RPEs are initiators of major pathological changes in many macular degenerative disorders, we believe that a better understanding of this complicated relationship between ER stress, autophagy and cell death may give us a novel perspective for restoring RPE-derived retinal degenerative diseases.

## Data Availability

The datasets created during and/or analyzed during the current study available from the corresponding author on reasonable request.

## Abbreviations

A2E: N-retinylidene-N-retinylethanolamine
AMD: Age-related macular degeneration
AMPK: Adenosine 5′-monophosphate activated protein
CHOP: CCAAT enhancer binding protein (C/EBP) homologous protein
ER: Endoplasmic reticulum
GRP78: Glucose-related protein 78
mTOR: Mammalian target of rapamycin
RNA: Ribonucleic acid
RPEs: Retinal pigment epithelium cells
